# Microfluidic Magnetic Mixing at Low Reynolds Numbers and in Stagnant Fluids

**DOI:** 10.3390/mi10110731

**Published:** 2019-10-29

**Authors:** Eriola-Sophia Shanko, Yoeri van de Burgt, Patrick D. Anderson, Jaap M. J. den Toonder

**Affiliations:** 1 Department of Mechanical Engineering, Microsystems Research Section, and Institute for Complex Molecular Systems (ICMS), Technische Universiteit Eindhoven, P.O. Box 513, 5600 MB Eindhoven, The Netherlands; e.shanko@tue.nl (E.-S.S.); Y.B.v.d.Burgt@tue.nl (Y.v.d.B.); 2 Department of Mechanical Engineering, Polymer Technology Research Section, and Institute for Complex Molecular Systems (ICMS), Technische Universiteit Eindhoven, P.O. Box 513, 5600 MB Eindhoven, The Netherlands; p.d.anderson@tue.nl

**Keywords:** microfluidics, magnetic micromixing, active and passive mixing, creeping flow

## Abstract

Microfluidic mixing becomes a necessity when thorough sample homogenization is required in small volumes of fluid, such as in lab-on-a-chip devices. For example, efficient mixing is extraordinarily challenging in capillary-filling microfluidic devices and in microchambers with stagnant fluids. To address this issue, specifically designed geometrical features can enhance the effect of diffusion and provide efficient mixing by inducing chaotic fluid flow. This scheme is known as “passive” mixing. In addition, when rapid and global mixing is essential, “active” mixing can be applied by exploiting an external source. In particular, magnetic mixing (where a magnetic field acts to stimulate mixing) shows great potential for high mixing efficiency. This method generally involves magnetic beads and external (or integrated) magnets for the creation of chaotic motion in the device. However, there is still plenty of room for exploiting the potential of magnetic beads for mixing applications. Therefore, this review article focuses on the advantages of magnetic bead mixing along with recommendations on improving mixing in low Reynolds number flows (*Re* ≤ 1) and in stagnant fluids.

## 1. Introduction

Lab-on-a-chip (LOC) devices integrate one or more miniaturized laboratory functions on a single chip. These devices are particularly interesting due to their small sample volume requirement, fast analysis, high precision control, versatility of the processes, and compactness of the systems. Many LOC concepts involve fluid transport and reactions, since most processes performed in LOC devices are for biological and chemical analyses. Consequently, microfluidics forms the backbone of LOC devices and deals with the manipulation and behavior of liquids at the scale of 1 µm up to 1 mm. Andreas Manz first introduced this approach for analytical chemistry [1], but the whole field of microfluidics significantly expanded when George Whitesides proposed an optically transparent polymer, Polydimethylsiloxane (PDMS), as a chip device material [2], making it easy to fabricate the chips and visualize the reagents within. Concrete recent examples of applications are detection of rare elements present in blood [3] or water [4].

Many LOC largely rely on chemical and/or biochemical reactions, often in parallel processes of assays with multiple reagents and samples. It is generally important to be able to achieve tunable and fast mixing in microfluidic devices that enhance these reactions (e.g., in the sub millisecond range [5]) in order to reduce the reaction time and speed up the sample analysis and process time [6]. For example, with respect to the detection and capturing of rare elements present in blood, it was found that the introduction of a mixing enhancement method using specific surface features (e.g., the herringbone mixer) in a microfluidic device led to an almost 80% capture efficiency of circulating tumor cells in a herringboned chip versus approximately 30% for a smooth walled one [7] indicating the increase of capturing elements due to mixed conditions. In general, the efficiency and reproducibility of capturing and detecting low concentration targets is strongly dependent on the global homogeneity of the reagents within the complete sample volume [8], which a mixing enhancement method can help achieve. Effectively, these enhancements methods can overcome limitations imposed by timescales of diffusion of targets (and possibly capture agents) over large distances and they maximize the chance that targets and capture agents meet.

There is a plethora of applications where micromixing is required (e.g., biomedical [9] or chemical [10]; biochemical [11] or pharmaceutical [12]). However, creating mixing in microfluidic devices is challenging since the Reynolds number *Re* is usually low in such small structures and, as such, inertial effects in the flow are negligible. *Re* is defined as:

*Re* = *ρvL*/*μ* (1)

where *ρ* is the density of the liquid, *v* the typical flow velocity, *L* the characteristic length and *µ* the dynamic viscosity.

In microfluidics, the length *L* and velocity *v* are relatively small. Therefore, the flow is always laminar and in the absence of turbulent chaotic flow, diffusion is the only mechanism to mix. However, diffusion is a slow and inefficient process at the typical scales of microfluidic channels of tens to hundreds of microns. A variety of methods have been proposed to tackle the challenge of mixing in microfluidics that speed up the effective molecular transfer. These mixing enhancement methods (called “micromixers”) can be divided into two categories: passive mixing and active mixing. Both types have previously been described [13] but a brief description is also provided below.

The passive micromixers employ smart geometrical designs to maximize the interface between the components. This type of micromixer makes use of two phenomena: molecular diffusion and chaotic advection. The former happens when fluids are in contact and exchange particles or molecules. Thus, it can be enhanced by increasing the interfacial area between the fluids. Chaotic advection, on the other hand, is explained as transport of elements in the flow driven by Langrangian dynamics, along chaotic and space-filling trajectories, creating topologies that exponentially increase a fluid-fluid interface [14]. One example of such a flow topology is the so-called “baker’s transformation”, which creates repetitive stretching and folding [15]. Chaotic advection can be achieved by placing obstacles in the stream path of the microfluidic channels. Since the exponential increase of interfacial area effectively enhances exchange by molecular diffusion, mixing is enhanced.

On the other hand, active micromixers use external forcing for inducing chaotic advection in the microfluidic channels or chambers. Many types of active micromixers have been studied, but their overall goal is to enhance mixing efficiency via introducing chaotic motion inside the chip, similar to passive mixers, but in a more controllable manner and potentially more effective. Pressure, temperature, acoustic forces, and Lorentz (magnetic) forces are some examples of phenomena that can be introduced.

A particular example of an active method that achieves high efficiency mixing is magnetic mixing. Magnetic mixing in microfluidics involves the usage of either magnetic microparticles (with diameters in the micrometer scale), the incorporation of ferro fluids (being colloidal suspensions of high concentration single-domain particles with typical dimensions of tens of nanometers in a liquid carrier [16]), or the use of magnetic microactuators for mixing purposes [17]. An external (electro) magnet manipulates such features.

Magnetic bead mixing focuses on the magnetic beads as a means of stirring fluids. A distinct feature of the magnetic beads is their ease of manipulation and control using an external electromagnet. They are also commercially available at a relatively low cost. Moreover, magnetic beads can offer additional features of capturing analytes/molecules [18] or isolating targets [19] by bio-functionalizing the magnetic beads’ surface. For example, antigen-coatedmagnetic beads have been employed as part of an ultrasensitive platform for the detection of biomolecules and proteins [20], whereas magnetic beads have been functionalized to selectively capture aptamers against cholera toxin [21]. A 2.5-fold improvement in biomarker capture for medical diagnostics was noted [22] while the formation of magnetic bead chains results in an almost 2-fold signal enhancement over the measured concentration range of a biosensor [23]. These cases exemplify the advantages of the magnetic beads used in medical diagnostics, showing that the magnetic bead functionalization capabilities and the magnetic properties for their separation from the sample using a simple magnet can be effectively combined.

In this review article, we focus on active mixing induced by magnetic means, using magnetic beads or magnetic actuators. In particular, we focus on situations with very low Reynolds numbers or stagnant fluids. This makes our review more focused than previous reviews of micromixing [6,9], but still relevant for a broad range of applications. In addition, the current article focuses only on the micromixing aspect of magnetic beads and actuators, where other reviews [8] analyze the general use of magnetic beads in bioassays.

To set the background, we start by discussing other means of micromixing. These methods are assessed by ease and cost of fabrication, their application at a very low Re number (*Re* << 1), their application in stagnant fluids (no-flow-through systems), their compatibility with biological components, the complexity of equipment or sample preparation steps required, and fluid rheology dependencies. Magnetic bead mixing is a good candidate for applications with said requirements. Magnetic beads can be utilized in soft magnetic structures such as magnetic artificial cilia (MAC), as well as magnetic bead chains as micro stirrers.

A brief introduction into the existing methods for mixing enhancement in microfluidic platforms, such as passive and active micromixers, will be given next. The review then continues with magnetic mixing as a choice method, with a particular focus on magnetic bead mixing. The article will conclude with a reflection on the future trend of magnetic micromixing.

## 2. Micromixing Methods

### 2.1. Passive Micromixers

**Figure 1:**
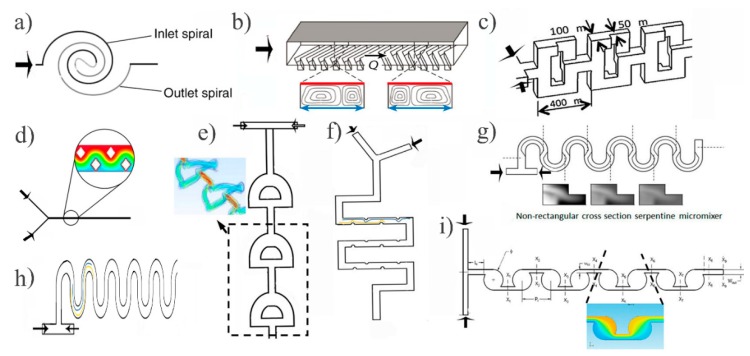
Various passive mixing enhancement designs. (**a**) Sketch of spiral microchannels; reproduced with permission from [29], published by the Royal Society of Chemistry, 2006. (**b**) The popular staggered herringbone; reproduced with permission from [41], published by The Royal Society, 2004. (**c**) The baker’s transformation; reproduced with permission from [42], published by Elsevier, 2017. (**d**) Example of obstacles placed in the fluid path stream; adapted with permission from [33], published by IOP Publishing, 2007. (**e**) The 3D “fishbone”-like design; adapted from [39] under a Creative Commons BY license, published by AIP, 2016. (**f**) Serpentine with added circular features inside the microchannel, (**g**) The 3D serpentine, adapted from [43] under a Creative Commons BY license, published by MDPI, 2018. (**h**) A simple serpentine structure. (**i**) A custom mixing design for *Re* = 1; reproduced with permission from [38], published by Springer Nature, 2017.

Passive micromixers make use of special geometries to mix and they are attractive due to their relative ease of fabrication and ease of integration into more complex LOC systems [24]. There are three main ways of passive mixing enhancements. That is either by placing an obstacle [25] or altering the layout of the microchannel [26], or a combination of these two. However, once built, they cannot be modified and therefore cannot be further optimized or tuned to a (different) specific need. Figure 1 shows some popular examples of passive micromixers published in the literature, including the popular staggered herringbone mixer (Figure 1b), serpentine structures (2D (Figure 1h), 3D (Figure 1g), and various other modifications. This pool of selection builds on the classical passive micromixer approaches such as the previously developed herringbone micromixer, but most of them (Figure 1b,c,e–g,i) are appealing to creeping flow based microfluidic applications. Spiral microchannels (Figure 1a) are mainly used for particle separation [27] or isolation [28] but they have also been assessed with respect to their mixing efficiency capabilities [29]. This type of mixing enhancement increases the mixing channel length while still restraining the chip surface area. This also causes additional mixing due to the Dean flows. Dean vortices occur due to the variation in the centrifugal forces and mixing occurs because of the velocity differences in the channel [30]. Nevertheless, spiral channel micromixing holds limitations at mixing Re ≤ 1 flows due to the weak Dean vortices [31]. On the other hand, placing obstacles in the channel perturbs the fluid stream path, creating lateral mass transport to improve mixing. This technique is dependent on the generation of eddies or turbulence [32], but in low Reynolds numbers this is hindered (48% mixing efficiency [33]). An example of obstacles placed in a microfluidic channel is seen in Figure 1d, but the mixing performance varies with obstacle shape [34].

A systematic breaking of symmetry in right-left helices in a herringbone mixer was demonstrated to yield better mixing [35]. Since then, all the staggered herringbones grooves (SHG) follow a similar approach (Figure 1b). For SHG, a mixing performance of ≥99% at *Re* = 1 at a mixing length of 5.8 mm was reported [36]. The SHG mixer is a good candidate for flow-through devices in applications that require a high degree of mixing within a relatively small mixing length.

Similar promising results were obtained by adopting a different type of passive micromixing design [37]. Comparing a basic serpentine (Figure 1h) structuring to one with additional semicircular obstacles of 150 µm radius along the microchannel (Figure 1f), yielded 100% mixing efficiency in both cases, but required shorter mixing lengths for the latter (14.4 mm versus 19.8 mm). The additional features become obstacles to the fluid path leading to chaotic advection. Yet, this method might be disadvantageous for small chip sizes because of the relatively long mixing length.

Possible design structures of passive micromixers are not limited to SHG and serpentine designs and finite element simulations form an excellent tool for evaluating the influence of different structures on the flow and resulting mixing efficiencies. Simulations of fluid flow in a mixer that included a number of circular mixing chambers (Figure 1i) yielded 99% mixing [38]. Hence, these numerical results should be experimentally evaluated to conclude to the design’s adeptness to good passive mixing.

A 3D “fishbone”-like design (Figure 1e) seems to be quite effective for *Re* low numbers due to resulting unstable fluid flow pattern [39]. Stretching, folding, splitting, or fluid instabilities in general, increase the fluid interface resulting in better mixing (Figure 1c). Compared to a single channel where such operations do not occur, a 3D mixer with sub-channels touring the fluid to branches of channels and then recombining them, is more effective for fluid manipulations, especially at very low *Re* (*Re* ≤ 1) [40].

The rheology of the fluid plays a highly important role for practical applications (e.g., mixing in commercial capillary-filling diagnostic platforms). The aforementioned micromixing studies have been performed in simple Newtonian fluids (like water), whilst biological fluids like blood or saliva are more complex and show non-Newtonian behavior. Saliva is a viscoelastic material with a strong shear-dependent viscosity [44], and blood shows non-Newtonian behavior at least in the length scales studied [45], whilst blood plasma exhibits Newtonian behavior in shear flow [46]. Thus, simple change of working fluid may provide different mixing performances in these micromixing methods [47] and the *Re* number formula for non-Newtonian fluids should be adjusted [48].

To conclude, passive micromixers do not require external forcing to induce local perturbations in the fluid that cause overall enhanced mixing and are typically suited only for low-viscosity fluids containing diffusive species. Often simple geometrical adaptations lead to sufficient mixing results. Nonetheless, it is evident that the mixing performance increases with the increase of axial length and in miniscule microfluidic platforms the luxury of space cannot be afforded. In addition, passive mixers are not controllable or adaptable after initial fabrication, making them less robust to changes in specific applications. Passive structures also require a certain footprint so there is a limit to how small they can be made. Perhaps most importantly, passive micromixers require flow generated by some pumping or filling mechanism, so they do not work for microfluidic chambers in which stagnant fluids (e.g., in a bioreactor) must be mixed.

### 2.2. Active Micromixers

Chaotic advection in fluids can also be achieved by externally inducing forces in the system. These forces lead to mechanical actuation, induced for example by ultrasound (acoustic mixing [49]), temperature (thermal mixing [50]), Lorentz forces (to create magneto-hydrodynamic flow or magnetic micromixing), velocity pulsing (pressure mixing [51]), or by applying non-uniform alternating electrical fields to fluidic solutions (dielectrophoretic micromixers [52]) to achieve fast and efficient microfluidic mixing. Other examples include electrohydrodynamic [53], time-pulsed microfluidic mixers [54] that apply electrokinetic driving forces to transport the sample fluids while simultaneously inducing periodic perturbations in the flow field and electrokinetic [55] (electrophoretic [56] or electroosmotic [57]).

**Figure 2:**
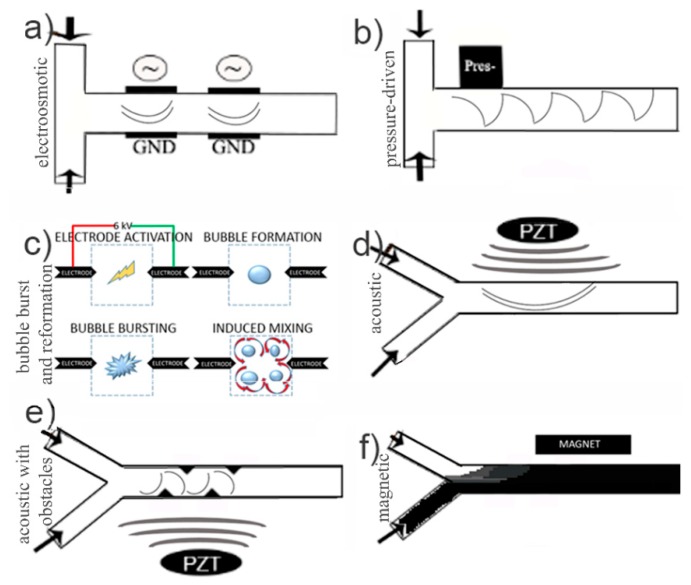
Active micromixing designs. (**a**) An electroosmotic micromixer with two electrode pairs. (**b**) A pressure driven microfluidic mixer. (**c**) The bubble burst-and-reformation based mixer, reproduced with permission from [69], published by Springer Nature, 2017. (**d**) Acoustic active micromixing. (**e**) Synergetic passive and active mixing mechanism. Solid triangular shaped blocks added in the path stream to contribute to an active mixer such as electroosmotic or acoustic. (**f**) Magnetic mixing with ferro fluids.

Active micromixers show good compliance with standard microfluidic requirements of small volume sampling and efficient mixing. In applications where the Reynolds number is very small (*Re* ≤ 1), as in some capillary driven microfluidics or stagnant fluids in microfluidic wells, active micromixers enable good mixing. Even though some remarkable work has been done in *Re* ≥ 1 mixing [58], some even reaching 100% mixing efficiency [59], not a lot of work has demonstrated on *Re* ≤ 1 systems or stagnant fluids. A number of active micromixer approaches proposed in the literature is seen in Figure 2 and evaluated in Table 1 by ease and cost of fabrication, their application for very low *Re* number, and/or stagnant fluids, compatibility with biological components, and complexity of required equipment or pre-steps.

The active electroosmotic mixers include electrodes on both sides of a microchannel. An increased mixing performance can be achieved by increasing the number of electrodes along the channel or increasing the applied voltage [60] (Figure 2a). Most electroosmotic mixers are only operational at low flow rates making the electrokinetic actuation of fluid mixing ideal for very low Reynolds numbers and/or stagnant flow. However, they hold other limitations. The increased number of electrodes increases fabrication costs and the increased voltage application can have detrimental effects such as fluid heating, especially in bio related applications. Therefore, specifically in low *Re*, non-biological specimens’ applications, electrokinetic driving could be a good candidate for mixing.

Electrical field gradients also generate local thermal gradients which result to a non-uniform thermal field; this thermal mapping may be a driving mechanism for mixing [61]; Electrothermal mixing has also been numerically investigated to generate efficient secondary flows that evoke mixing of 83% in a pretreated ionic solution [62]. Electrothermal microfluidic flow has also been actuated by light where the mixing index was observed to increase by 65% within three seconds of the actuation mechanism [63]. When environmental stability is not essential, temperature driven micromixers have been studied but their mixing performance is not ideal for stagnant fluids. This type of mixing mechanisms exploits temperature gradients [64], thermal bubbles [65] or thermo-viscous expansion-contraction effects to enhance mixing in microchannels [66].

Pressure-driven micromixing mechanisms employ pneumatic pumps [67] (Figure 2b) or different means of perturbing the fluid motion based on pressure driven oscillatory flow [68]. In contrast to electrokinetic mixers, pressure induced fluid perturbations can be applied to micromixing in microreaction chambers (e.g., bioreactors) with biological components. They can also be used for mixing no-flow-through systems.

A different active mixing mechanism is the use of cavitation bubbles. The formation and bursting of bubbles inside a fluid can lead to mixing in microfluidic channels. Up until now, this scheme has been limited by the cost of equipment needed for the bubble formation (e.g., high-power lasers). However, a portable battery-powered electric circuit was developed [69] to create an electric spark between two electrode tips and generate cavitation events (Figure 2c), which in turn resulted in flow disturbances. The continuous bubble formation and collapsing led to a 98% mixing efficiency in milliseconds in a 200 µm mixing channel. This method still bears the same disadvantages as the electrokinetic mechanisms, since it requires a pre-step of adjusting the conductivity of the fluids with phosphate-buffered saline (PBS). However, this is not the only setback. A repeated abrupt electric discharge of 6 kV for 25 µs is not only bio-cell non-friendly, but also generates heating effects inside a microfluidic channel of these small volumes.

Acoustic actuation (Figure 2d) incorporates one or more sound wave generating transducers to introduce chaotic fluid behavior in a microchannel or a microreaction chamber [70]. These waves travel by compressing and decompressing the fluid. This type of actuation has demonstrated great mixing performances, e.g., utilizing localized ultrahigh frequency (UHF) acoustic fields that result in 87% homogeneous mixing within 1 ms [71] or combined with sharp edged structures [72] (Figure 2e). These performances take advantage of passive mixing enhancement methods with the introduction of obstacles to the fluid lamellae. The feature of a synergetic passive (solid blocks in Figure 2e being obstacles to the fluid stream) and the active mixing mechanism has also been studied with electroosmotic mixers [73]. In general, acoustic energy is of mechanical nature in the form of vibrations and pressure fluctuations. Thus, one has to consider these effects on biological cells, especially in terms of influence to the cell’s mechanical environment due to the induced pressures [74]. Acoustic actuation techniques are used for lysis of cells for DNA extraction [75], but they also hold limitations since prior treatment for the weakening of the cell membrane has been required [76]. In addition, it should be recognized that both temperature induced mixing and acoustic actuation raise concerns in microfluidic systems that involve biochemical reactions where temperature should be controlled cause potential heating issues that might damage the sample. However, heating effects induced by acoustic actuation are strongly dependent on the frequency and type of acoustic field, and these can both be controlled [77].

A particular interesting mechanism is based on magnetic forces that can be translated into fluid motion. Magnetism offers the advantage of combining biocompatibility with excellent mixing. Such a simple experimental scheme, based on the use of ferro fluids, is seen in Figure 2f [78]. However, if magnetic gradients are desired, the orientation of the permanent magnet can be adjusted by simply placing the magnet perpendicular to the microchannel [79]. This solution demonstrates similar mixing results at lower *Re* numbers and smaller dimensions but with a perpendicular arrangement of the permanent magnet to the microfluidic channel, resulting in a non-uniform magnetic field, to induce gradient micromixing.

In Table 1, an overview is given of the different mixing mechanisms and their advantages and disadvantages. Notably, to assess whether a mixing device for a specific method is “easy to fabricate”, we evaluated whether it requires many processing or assembly steps or if fabrication methods are not easily accessible (e.g., advanced cleanroom processes). To assess whether a method is suitable for “low cost fabrication” we additionally evaluated whether high cost materials (e.g., piezoelectric) are needed.

In general, active micromixers are often more complex to fabricate (e.g., acoustic micromixers) due to their complex structures and their need for external components (e.g., power sources) to operate. However, mixing in these devices can be tuned for providing optimal mixing efficiencies. Active mixing mechanisms can overcome the mixing challenges in non-Newtonian fluids [80] makes them superb for commercial diagnostic platforms. Moreover, active micromixing does not require existent fluid flow to work, and can therefore be applied to microfluidic chambers with stagnant fluids. Micromagneto fluidics in microfluidic systems have extensively been studied [81], with some reaching mixing efficiencies >95% even within 2.0 s at a distance 0.3 mm downstream of the mixing channel [82]. However, the densely concentrated ferrofluid might hinder optical readouts (as illustrated in Figure 2f). To tackle this issue, magnetic micromixing with magnetic beads arranged in chains can be used.

Finally, most active micromixing devices presented in the literature are manufactured from PDMS using soft lithography. This approach is very suitable for research and development purposes, but towards industrial application this is not the best approach since the manufacturing is relatively expensive and slow, in addition to the fact that PDMS absorbs small molecules is a disadvantage for biomedical applications. However, the mentioned micromixing concepts can also be realized using more suitable materials and fabrication approaches such as injecting molding on thermoplastics. In addition, some of the methods require the inclusion of patterned electrodes (i.e., electroosmotic mixing, cavitation bubble mixing) or piezoelectric materials (i.e., acoustic mixing), which results in higher fabrication costs.

### 2.3. Active Micromixing Applications

As previously noted, stagnant fluids in microreaction chambers (no-flow-through microwells) are difficult to mix, simply because of the lack of mass transfer [83]. However, in certain applications in which thorough mixing is necessary, this challenge should be addressed. An example to this is the microbioreactor. The microbioreactor consists of a microwell where biological elements (cells or bacteria) are usually placed and a surrounding channel that allows for flowing nutrients (i.e., oxygen) required for the proliferation of the biological elements. These two components are connected with a porous membrane (usually PDMS) to accommodate for the transfer of nutrients to the microwell [84]. The challenge arises when the biological elements located to points furthest from the nutrient flowing channel are not cultured as effectively as their neighboring elements closer to the channel are. It has, thus, been found that mixing enhancement mechanisms enable fast and adequate oxygen transfer in bacteria cultures in microbioreactors [85]. In a similar scenario, the static mixer has been used in 24-well plate to find that cell proliferation was improved with a significantly higher specific growth rate in round wells [86].

Another distinct example is the usage of magnetic beads as carriers for the development of assays, i.e., the enzyme-linked immunosorbent assay (ELISA). ELISA is a plate-based assay technique designed for detecting and quantifying antibodies or proteins. ELISAs are typically performed in well plates but microfluidic approaches have also been investigated [87]. In a bubble-driven micromixer within a microreaction well the detection of a biomarker in bladder cancer patients’ urine only required 30 to 40 min compared with the 3 to 4 h required for a conventional ELISA [88].

## 3. Mechanisms of Magnetic Mixing

Several types of magnetic mixing mechanisms exist, and they all share the fact that the magnetic actuation is done from outside the chip with an external magnet. Magnetically induced micromixing is a viable choice for a wide variety of microfluidic applications due to the obtained high mixing efficiencies, biocompatibility, little need of complex equipment/ fabrication, and their effectiveness at *Re* ≤ 1 or in microreaction chambers with stagnant fluids. Two main examples are magnetic artificial cilia and magnetic bead chains.

### Magnetic Artificial Cilia

Cilia in nature are slim, hair-like protrusions of cells that function to generate propulsion, induce flow, mix solutions, and transport species such as cells or debris in a no-flow-through microwell [89]. Therefore, artificial cilia are microscopic microactuators inspired by nature. There are various ways of actuating artificial cilia (e.g., acoustically, electrically, by light, etc. [90,91]), but herein we focus on the magnetically actuated artificial cilia (MAC). MAC usually consists of ferromagnetic or superparamagnetic particles dispersed in a magnetically inactive material, often in PDMS due to its inherent flexibility and simple fabrication methods.

Theoretical studies have taken place both on the cilia movement [92] and the beating kinematics [93]. Once an electromagnetic field is induced inside a microwell [94] or not [95], they can be actuated either simultaneously [96,97] or individually [98] for a closer mimicking of the biological cilia. It was also demonstrated that alternating and asymmetric actuation patterns [99] of cilia movement (beating and rotational motion) can induce mixing by chaotic advection [100]. As such, artificial cilia have potential for mixing purposes.

Magnetic actuation on the other hand is in practice relatively easy to realize since a rotating permanent magnet would suffice to circularly move the MAC and not interfere with the functions of biological specimens. Examples of MAC in fluids of *Re* ≤ 1 and/or microchambers for fluid mixing are shown in Table 2.

**Figure 3:**
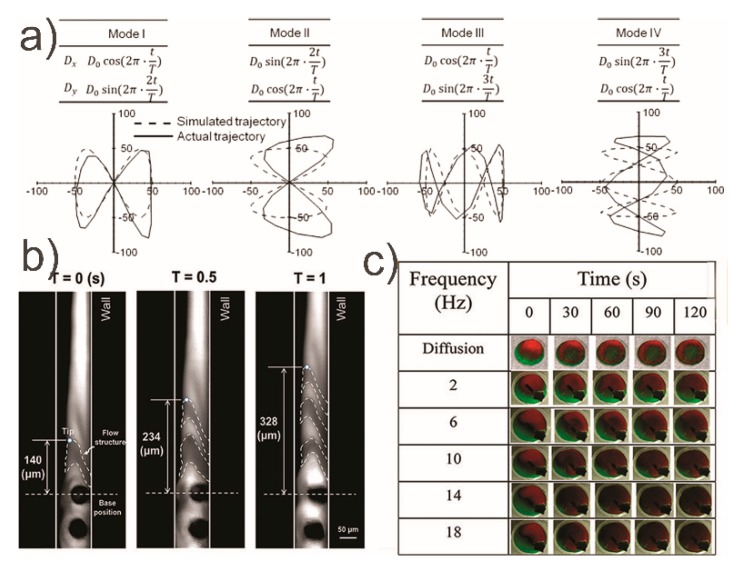
Results from the work depicted in Table 2. (**a**) The investigation of the dynamics of four beating configurations; reproduced with permission from [101], published by Elsevier, 2016. (**b**) Time lapse imaging of the flow induced by the vertical conical motion; reproduced from [102] under a Creative Commons BY license, published by MDPI, 2018., (**c**) Mixing by the cilium in a fluidic chamber for different times and frequencies; reproduced with permission from [104], published by IOP Science, 2016.

To demonstrate the efficacy of MAC for mixing, 50 µm wide and 400 µm long MAC were fabricated to move at a low magnetic field strength of 170 mT [101]. The authors investigated four beating configurations of the symmetrical and asymmetrical vertical motion of their cilia (Figure 3a) and measured mixing. Mixing of the two Newtonian glycerol-based solutions in their experiment reached 91%. Using a similar cilia formation but with a simplified vertical conical motion, an 84% mixing efficiency was achieved [102] (Figure 3b).

At the millimeter scale, a magnetically actuated cilium under a rotating field can act as a micro stirrer in a fluidic chamber of higher dimensions and still obtain decent mixing efficiency of 80% in larger volumes [104] (Figure 3c). In this case, there was only one cilium under an 18 Hz magnetic driving force.

**Figure 4:**
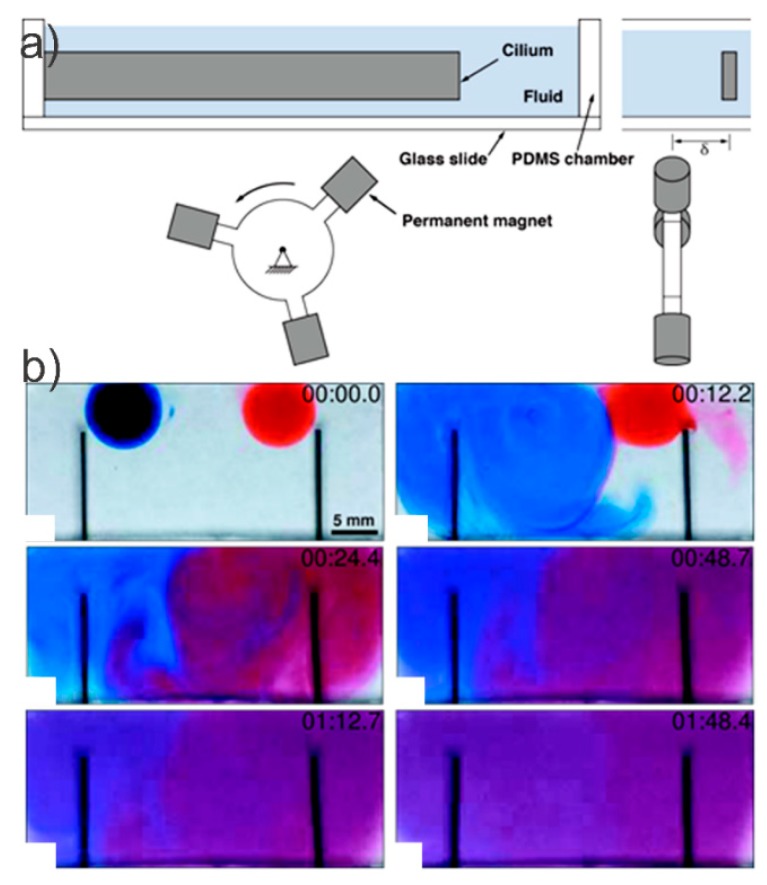
The experimental setup and results of [103] for individually controlling two cilia in a fluid chamber. (**a**) The setup configuration and (**b**) time lapse images with the best performed alternate pattern cilia beat. Reproduced with permission from [103], published by the ASME, 2017.

Two individually controlled magnetic cilia for mixing (Figure 4) were produced to investigate mixing [103]. It was found that in contrast to simultaneous activation, the alternate activation of the two cilia inside the fluidic microwell created two spatially overlapping vortices resulting in better mixing (Figure 4b). In fact, the results of both [103,104] are indicative of the potential of magnetic cilia for mixing no-flow-through systems (microwells). However, one has to consider that these results were based on relatively long cilia (3–6 mm and 13 mm, respectively), which is less useful for most microfluidic applications with small size requirement (micron scale).

On the other hand, it is difficult to individually control cilia in a more miniaturized system. To overcome this sizing limitation, it was shown that simultaneous actuation can initiate tilted conical motion, which induces a flow speed of 120 μm/s in the center of a much smaller recirculation channel [105]. This may be exploited for mixing at sub-mm scales.

Substantial flow velocities (260 μm/s) in a circular channel of a rectangular cross section with a height of 900 μm and a width of 5 mm were reached [106]. For mixing to be effective, fluid circulated by the cilia must sweep the full microreaction chamber in a reasonable time. In cilia, the generated fluid velocity is dependent on two parameters, the bending angle of the tilt of the cilium, and the opening of the conical cilia motion. A fabrication technique to produce cilia that can reach a bending angle of 72° instead of 45° of typical cilia found in literature has been proposed [106]. Even though this work focusses on fluid pumping, this higher angle may be beneficial for mixing purposes in microfluidics, too. Should the width and height of the chamber decrease, generation of flows in stagnant liquids, and subsequent efficient mixing with MAC may be achieved with a cilia length of 350 μm [106].

In addition, chaotic advection must also develop in the fluid; mixing is certain to be poor unless flow in the microreaction chamber is chaotic. This chaotic motion may be introduced by adding passive structures on top of the cilia region.

Currently MAC have been used mainly for pumping purposes, but can also be applied for mixing, considering the large flow speeds that can be generated. Especially in applications employing only a microchamber without external flow. It would thus be interesting to evaluate these options for mixing. Nonetheless, it is recognized that the fluid perturbations generated in cilia are very much dependent on the cilia length and the channel dimensions. Magnetic beads, on the other hand, do not bare this restriction since they are very small and can be moved freely in 3D, exhibiting collective motion and potentially resulting in high fluid velocities and perturbations.

## 4. Magnetic Mixing Induced by Magnetic Bead Chains

Suspended magnetic beads can self-assemble into chains due to dipole-dipole interactions [107]. The chains can be either attached to the floor of the microfluidic chip [23] or remain freely in the volume of a no-flow-through microchamber [108]. Magnetic mixing can then be achieved by employing external electromagnets for the introduction of a rotating magnetic field.

### Rotational Behavior of Magnetic Bead Chains and Effect on Mixing

Superparamagnetic beads are usually spherical and consist of ferromagnetic single-domain nano-elements incorporated in a polymer matrix and can be actuated with external magnetic fields. These beads move along the gradient of the field induced by a magnet, either an electromagnet, a permanent magnet or a hybrid (e.g., a static gradient magnetic field and an external AC uniform magnetic field [109]). Because of dipolar attractive interaction when in the presence of a unidirectional magnetic field, beads form chains. This chain formation leads to shape anisotropy and thus magnetic torque can be applied on a superparamagnetic body if its shape is anisotropic. The magnetic bead chains can be made to rotate by rotating the magnetic field and as a result they can be used as micro stirrers and induce local micromixing in LOC devices.

**Figure 5:**
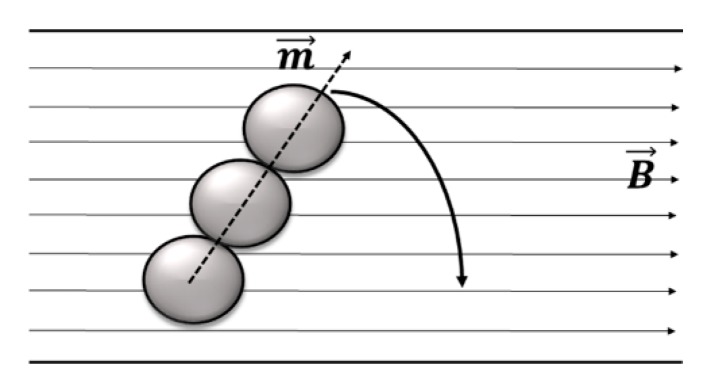
Side view drawing of a magnetic bead chain with moment $\vec{m}$, trying to align to an applied magnetic field $\vec{B}$ in the axial direction.

The torque (τ) acting on a magnetic bead chain is caused by the attempt of the chain to align to the magnetic field, as shown in Figure 5, in which $\vec{m}$ is the magnetic moment of the chain. It has also been proven that only the outer magnetic beads in a chain have a net contribution to the driven magnetic torque [110], but the precise description is out of the scope of this review. Under a rotating magnetic field, this tendency to align to the field lines will result in the continuous rotational movement of a magnetic bead chain.

The magnetic torque is expressed by the following equation:

(2) $$\vec{\tau }=\;\vec{m}\;\times \;\vec{B}$$

**Figure 6:**
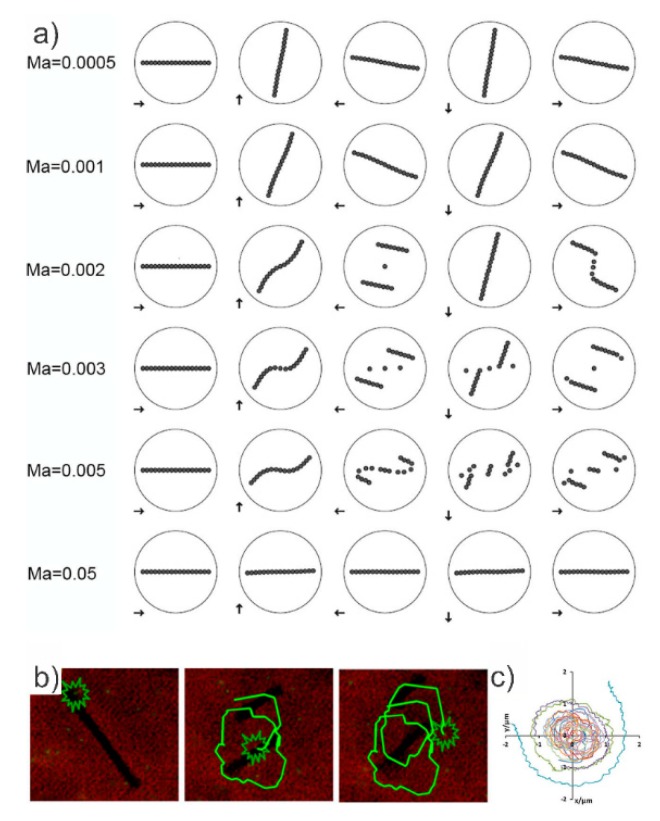
(**a**) Dynamics of the chain at six Mason numbers. The arrows depict the magnetic field directionality; image reproduced with permission from [112] published by the American Physical Society, 2007. (**b**) Particle tracing results obtained for periodic chain breaking and chain reformation. The induced motion of a tracer particle following the fluid at different time steps [116]. (**c**) The overall trajectories of the tracer particles, starting at different radial distances from the center (0, 0) [116]. (**b**,**c**) reproduced with permission from [116], published by Springer Nature, 2013.

A key parameter that determines the dynamics of the rotating chain is given by the dimensionless Mason number (Ma), defined as [111]:

(3) $$Ma=\frac{16\eta \omega }{\mu_{0}\chi^{2}H^{2}}$$

where *η* is the viscosity of the surrounding fluid, *ω* is the angular velocity of the magnetic field, χ the dimensionless susceptibility of the beads, *H* the field strength and $\mu_{0}$ the permeability of free space equal to 4π × 10^−7^ N/A^2^. The Mason number represents the ratio of viscous forces to magnetic forces acting on the beads. The motion of the chain, fluid flow, and mixing are significantly influenced by the Mason number [112] (Figure 6a). When the magnetic forces are dominant, at low Ma (Ma < 0.001), the chain will rotate like a rigid or deformed rod at a constant angular velocity synchronous with the period of the external field. In order to overcome viscous drag, the chain may rotate with increasing phase lag θ from the external field, thus increasing the magnetic torque to balance. Under increasing value of the Mason number (0.001 < Ma <0.01) viscous drag increases relative to the magnetic interactions among the particles, therefore chain breakup is observed. At around Ma = 0.002, the magnetic bead chains break up and reform in an alternating manner whilst in higher Mason numbers (Ma ≥ 0.01) the drag forces become dominant therefore the chains do not reform [112], but on average the chains rotate with a frequency lower than that of the rotating field.

To make the magnetic bead chains more flexible and less prone to breaking up, the beads can be linked with DNA [113] or PEG [114]. In this way elastic forces are introduced. Rotational dynamics of magnetic bead chains have been experimentally and numerically investigated [113] along with the magnetic bead chains’ velocities and the fluid velocities they subsequently induce [115].

It has been computationally found that the local mixing efficiency is greatly enhanced, when the chains, during rotation, repetitively break up and reform, rather than rotating as rigid rods [116].

The chain break is not only dependent on the Mason number but also the chain length. A novel dimensionless parameter *R_T_*, including both effects of the Mason number and length of the chain has been derived to characterize rotational bead chain dynamics [110]:

(4) $$R_{T}=16\frac{\eta \omega }{\mu_{0}\chi_{\varrho }^{2}H_{0}^{2}}\frac{N^{3}}{\left(N-1\right)\left(\ln\left(\frac{N}{2}\right)+\frac{2.4}{N}\right)}$$

where *N* is the number of particles forming the chain. Rather than representing the ratio of forces, like Ma, *R_T_* represents the ratio of viscous to magnetic torques. If *R_T_* < 1 the chain rotates as a rigid rod following the field. If *R_T_* > 1, the chain breaks up and possibly reforms. However, this break up and reformation significantly compromises the motion efficiency of oscillating microchains [117]. This means that the chain may rupture and fail to reform again. The efficiency of chain reformation has also been studied [118].

The effect of the magnetic bead chains on the surrounding fluid inside a no-flow-through microwell if in a rotating magnetic field was next analyzed [116]. Ideally, one would combine many electromagnets to get a field that is as controllable and smooth as possible. A setup employing eight electromagnets for an accurate and robust 3D control of the magnetic bead chains—4 electromagnets in one plane as in and another set of four electromagnets in a plane perpendicular to the first plane has been constructed [108]. Figure 6b shows the results in the case the chain periodically fragments and reforms where the fluid was visualized with the introduction of passive fluorescent microbeads for trajectory acquisition [116]. Figure 6c clearly shows the effect of the break up and reformation of magnetic bead chains on the surrounding fluid. It is obvious, that the effect is mainly local around the edges of the magnetic bead chain, i.e., local mixing occurs. Frequency of rotation plays an important role in micromixing as well. At higher frequencies (*R_T_* >> 1), the bead chains will experience larger viscous drag forces and collapse to smaller structures that can continue to rotate individually. Since the periodic break up and reformation of the magnetic bead chains is what enhances mixing, *R_T_* ≈ 1 is desired. Even though work on micromixing inside a no-flow-through microchamber using flexible bead chains has taken place [119], a comparison between the mixing efficiency obtained from the flexible chains and the mixing efficiency obtained from the chains collapsing and reforming has yet to be done.

**Figure 7:**
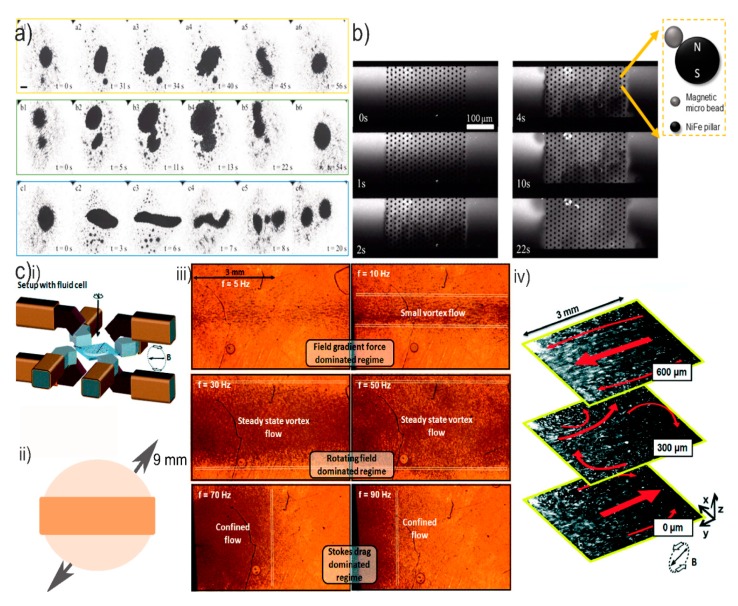
(**a**) Experimental results at a 7.5 mT rotating magnetic field. (**a1**–**a6**) Reversible spread state (yellow square), (**b1**–**b6**) merging of two swarm vortexes (green square), and (**c1**–**c6**) splitting of a swarm vortex to two smaller swarms (blue square). Scale bar 600 μm. Image reproduced with permission from [120], published by SAGE, 2018. (**b**) The NiFe array of [122]. Magnetic beads were attracted to each NiFe pillar and once the magnetic field becomes rotational, the magnetic beads started rotating by locally stirring the fluid. Since the microchannel floor consisted of a planar array of several NiFe features, the collective rotation of all pillars resulted in global mixing. Images reproduced with permission from [122], published by Elsevier, 2016. (**c**) Global collective motion behavior of micro stirrers [123]: (**i**) The experimental setup of the chamber in the middle of an octopolar electromagnetic system, (**ii**) top view sketch of the microfluidic chamber with an indication of where the vortex occured, and (**iii**) the different regimes of swarming particles. A clockwise rotating vertical magnetic field (30 mT) was applied and the final shape of swarming particles was observed at different rotational frequencies of the applied field. A steady state vortex flow stretched along the entire length of the microchamber, was found at 30 Hz and 50 Hz. (**iv**) Flow behavior. The global induced motion (red arrows) of the fluorescent tracer particles (white spots in three planes over the height of the cell. Near the bottom (z = 0 μm) and upper (z = 600 μm) surfaces of the fluid cell, the flow field was unidirectional. At the center of the cell (z = 300 μm), small and local vortices appeared. Images reproduced with permission from [123], published by the Royal Society of Chemistry, 2015.

Using a rotating magnetic field on paramagnetic beads, an intriguing phenomenon of vortex flows inside a no-flow-through microchamber was observed [120]. The vortex induced by a rotating paramagnetic nanoparticle chain exerted long-range attracting interaction forces on adjacent chains that gradually minimized the distances between the chains. When the distance of two rotating chains (two induced vortices) became smaller than a critical value [121], the chains performed coaxial rotation and the two vortices merged resulting in strong vortexes. The particle chains rotated around themselves but simultaneously rotated about the center of the vortex due to the dynamic balance of the radial components of the interaction forces (Figure 7a). Although the specific application that was targeted in this study involved drug delivery, this approach can also be beneficial for mixing. The magnetic chains not only rotate around themselves (local mixing) but also rotate in a global motion depicting collectivity. Moreover, the merging and splitting of the swarm vortexes (b1–b6 and c1–c6 in Figure 7a) may cause chaotic effects in the surrounding fluid therefore contributing potentially to mixing.

To avoid a sophisticated experimental setup, a method to orbit magnetic beads around using easily microfabricated circular permalloy (NiFe) pillars on the floor of a microfluidic channel was presented [122] (Figure 7b). This in turn led to rapid mixing in a 150 µm wide and 270 µm long array with a mixing index of almost 70%, but it is believed that mixing can be enhanced if the NiFe array consists of more NiFe features.

In a different configuration, global (9 mm) collective motion of the rotating magnetic bead chains was observed with specific magnetic field rotation settings [123]. The magnetic bead chains rotate around themselves (local mixing) but also depict global mixing (iii in Figure 7c). At certain rotational frequencies, combined with certain configurations for the magnetic field (different electromagnet protocols of ii in Figure 7c), the phenomenon occurs: a global vortex [123], but not limited to the central axis of the swarm vortex as in [120]. This favors chaotic mixing, as noticed in iv in Figure 7c.

However, as illustrated in ii in Figure 7c, this motion of the rotation magnetic bead chains (along the horizontal axis of the whole chamber) is limited to a certain band of the total microfluidic area. Therefore, full chaotic global mixing is not yet obtained. One can imagine that a different chamber shape might have an effect on the global mixing efficiency. Alternatively, a magnetic actuation protocol that can drive the magnetic bead chains along the whole microfluidic area may provide great mixing efficiencies.

In recent work, magnetic nanobars effectively stir small volumes in micro droplets [124]. Perhaps in the same configuration of the magnetic experimental setup [123] a swarm of rotating nanobars in a no-flow-through chamber can mix fluids, too. In fact, similar collective motion of magnetic nanoparticles, as depicted in Figure 7a,c, has been reported, although its resulting mixing efficiencies were not discussed [125]. In earlier work, a single and collective motion of magnetic micro swimmers was studied [126] along with clusters as governed by their hydrodynamic and dipolar interactions [127]. However, artificial micro swimmers have not been evaluated as mixing mechanisms in the aforementioned work. Artificial micro swimmers are features inspired by nature and they have been known to induce mixing of the surrounding fluid during locomotion [127]. Therefore, it would be interesting to determine whether a collective motion of artificial magnetic micro swimmers would be beneficial for mixing.

Although dynamic behavior of superparamagnetic beads under the influence of rotating magnetic fields and its immediate effect on the surrounding fluid have been numerically and experimentally studied, not much research has been done on the collective motion of the micro stirrers and its effect on the mixing efficiency in microfluidic chambers or channels. As such, there is a lot of room for research into global mixing efficiency induced by the collective magnetic bead chain micro stirring mechanisms.

## 5. Outlook on Magnetic Mixing

There has been great work conducted that employs magnetic beads for both microfluidic mixing as well as for capturing and detecting analytes [128], as well as for the formation of complex immunoassays [129]. It has been proven that magnetic bead chain rotation, break up, reformation, and the collective behavior of micro stirrers can provide the chaotic flow that leads to diffusion enhancement, in addition to efficient mixing. However, in applications where the full rotation of magnetic bead chains is demanded, the requirement of several external magnets for the precise beads’ manipulation in the microfluidic chip can also become disadvantageous. In turn, this demands a combination of at least three electromagnets and an AC signal generator for the wave application to the coils. In this general approach, electromagnets can be placed external to the microfluidic device and the device itself can be relatively simple. The choice of materials for fabricating the device is then wide, as long as the material is non-magnetic. That means that, next to PDMS, which is often used for proof-of-concept experiments, for eventual products also more suitable materials and fabrication method can be used, such as injection molding of thermoplastics.

Alternatively, if even more control is needed, microcoils and electrodes can be integrated in chips to locally produce a magnetic field [130]. Such microelectromagnets-on-chip configurations enable the generation of precise magnetic fields and field gradients within the chip. An example of this are two planar spiral microcoils perfectly aligned to the top and the bottom of the microfluidic channel for the detection and quantification of superparamagnetic beads based on the frequency mixing technique [131]. In contrast to external larger coils that are prone to error due to human placement, the alignment in microelectromagnets-on-chip occurs in the fabrication process. Since the generation of a magnetic field is strongly related to the current applied to the micro coils, it is an important parameter to consider. If a high magnetic field strength is required for the application, potentially high currents pass through the micro coils, which can lead to overheating (i.e., micro fluid boiling) [132], which causes substantial damage to biological samples or even the chip itself. As such, single [133] or array-based [134] micro electromagnets are favorable to facilitate low magnetic strength conditions. Another disadvantage is that the incorporation of microelectromagnets-on-chip might be considered too demanding due to the fabrication processes.

If simple and fast fabrication is required, additional PDMS-based magnetic strips in microfluidic devices could lead to enhanced magnetic cell separation [135]. An additional external rotating magnetic field can also be added for further mixing enhancement. In fact, integrating mushroom-like soft magnetic structures underneath microfluidic chambers was demonstrated to result in magnetic bead transportation according to a mechanism similar to that of a conveyor belt in a no-flow-through system [136]. This involved combining an external rotating magnetic field and the magnetic structures. A mixing chamber can be envisioned with rotating agglomerates where the local magnetic forces drive the magnetic beads to desired locations.

A different approach can involve combining active mixing with passive components. Such hybrid passive and active micromixers are expected to further enhance mixing. In fact, recent work on ferrofluids in a T-shaped microfluidic device [137] has taken place reaching a mixing efficiency of 97.5%. This is an early indication that a hybrid approach could give synergetic results. An interesting suggestion would be to combine passive SHG-like features with magnetic bead chain micromixing or MAC, which may lead to a synergistic effect of two approaches that have already been proven separately. This can be further extended to more combinations of passive and active mixers.

The magnetic beads themselves can also be changed and their dipole-dipole interactions can be exploited to induce better mixing schemes. Magnetic bead parameters such as iron content, size, shape, and chemical composition can be altered. Different magnetic bead shapes, other than spherical, exhibit anisotropy shape [136]. Different chemical magnetic composition can offer stronger dipole-dipole interactions. Highly tunable synthetic antiferromagnetic platelets have been fabricated by their controlled actuation in an applied magnetic field that can locally exert large torques [138]. The potential micromixing effect of this technology is that the platelets can either chain up in a planar arrangement under a certain magnetic field (one side of a platelet to a neighboring one) or in a vertical arrangement (one’s platelet surface to another’s) in a higher magnetic field. However, whether the formed chains in a rotating magnetic field will rotate to mix the fluid still needs to be investigated. An even bigger question arises on their collective rotational movement along a microfluidic chamber to induce global mixing.

Although there are many advantages, magnetic micromixing also has disadvantages and limitations. First, the need for a magnetic actuation system based on permanent magnets or electromagnets, somewhat complicates the complete system, but this becomes a bigger limitation when integrated electromagnets are required since, as mentioned above, this leads to increased fabrication cost and possible heating effects. Second, the presence of magnetic beads in the microfluidic device might hamper optical read-out that is often used in biochemical assays; this can be avoided by moving the magnetic beads out-of-sight using a specific magnetic field. Third, the sheer presence of the mobile magnetic beads might interfere with other processes in the chip if the beads themselves do not participate in the biochemical assay.

The latter disadvantage of free magnetic beads is resolved by magnetic artificial cilia that, being connected to the walls of the microfluidic chip, form an integral part of the device. The artificial cilia, next to induce mixing, can be used also to pump liquids or even to transport particles or cells in microfluidic devices [139]. Therefore, magnetic artificial cilia offer the perspective of versatile use in microfluidic chips beyond micromixing. The attachment of the cilia to the walls forms at the same time a limitation since 3D mixing may be more difficult. Combining artificial cilia mixing with other approaches (e.g., passive mixing structures) may form a way to enhance the mixing efficiency in that case.

From recent developments it becomes clear that a wide range of possibilities using magnetic micromixing in microfluidics can be envisioned. The electromagnets can be minimized on chip to control gradients and additional passive soft magnetic structures can be added to aid magnetic bead or artificial cilia movement. Furthermore, a combinational approach of a hybrid passive micromixing with active mixing components can be envisioned. Finally, properties of magnetic beads as well as artificial cilia can be tuned to fit micromixing requirements either in shape and size or chemical composition by adjusting their magnetic properties.

## 6. Conclusions

In contrast to passive micromixing strategies, magnetic beads and artificial cilia offer an advantage that no external flow is required for their actuation and induce fluid mixing. This makes them beneficial to diagnostic systems that require capillary filling (reducing the cost/complexity of additional microfluidic pumps and valves) or involve stagnant fluids within microchamber reactors. In addition, for applications that require the capturing of analytes, the magnetic beads offer the possibility of applying a functional coating based on antibodies [140]. These coatings can also prevent aggregation (e.g., carboxylic group coating).

Extensive work has been done to tackle the mixing challenges in microfluidic devices, resulting in diverse and creative solutions. Still, there is plenty research to be done, all the more because the number of microfluidic applications has exploded in the past decade. Magnetic mixing shows favorable results in mixing efficiencies. However, it seems that there is no single mixing concept fulfilling all the requirements set by the application envisaged [141]. In terms of small microchambers with no flow, we have suggested a number of possible alternatives using magnetic mixing, either by microfabricating techniques where microcoils are incorporated in the microfluidic chips, or by designating passive soft magnetic components to the steer magnetic bead movement. Finally, synergetic active and passive mixing and tuning magnetic bead parameters might enhance micromixing.

## Figures and Tables

**Table 1:** Comparison of the different mixing mechanisms.

Mixing Mechanisms	Electro Osmotic	Pressure-Driven	Temperature-Driven	Acoustic	Cavitation Bubbles	Magnetofluids
Easy to fabricate	N	N	Y	N	N	Y
Low cost fabrication	N	N	Y	N	N	Y
Applicable to very low *Re* numbers	Y	Y	Y	Y	Y	Y
Applicable for stagnant flows	Y	Y	N	Y	Y	Y
Compatible with biological components	N	Y	N	N	N	Y
Sample preparation needed	Y	N	N	N	Y	N
Readout visibility	Y	Y	Y	Y	Y	N

Y/Yellow: Yes and N/Blue: No.

**Table 2:** Magnetically actuated cilia (MAC) examples of *Re* ≤ 1 over the past few years.

MAC for Fluid Mixing					
Reference	Mixing Mechanism	Experimental (E)/ Simulation (S)	Overall Fluidic Dimensions	*Re* Number	Mixing Efficiency (%)
[101]	MAC	E and S	*H* = 400 µm, *W* = 114 µm, *L* = 152 µm	3.18 × 10^−3^	91
[102]	MAC	E and S	*H* = 500 µm, *W* = 100 µm, *L* = 500 µm	1.16 × 10^−3^	84
[103]	MAC	E	*H* = 4 mm, *D* = 5 mm	microwell	80

*H* = Height, *W* = Width, *L* = Length, *D* = Diameter.
